# In the Search of Potential Serodiagnostic Proteins to Discriminate Between Acute and Chronic Q Fever in Humans. Some Promising Outcomes

**DOI:** 10.3389/fcimb.2020.557027

**Published:** 2020-09-18

**Authors:** Anna Psaroulaki, Eirini Mathioudaki, Iosif Vranakis, Dimosthenis Chochlakis, Emmanouil Yachnakis, Sofia Kokkini, Hao Xie, Georgios Tsiotis

**Affiliations:** ^1^Department of Clinical Microbiology and Microbial Pathogenesis, School of Medicine, University of Crete, Heraklion, Greece; ^2^Laboratory of Biochemistry, Department of Chemistry, School of Science and Engineering, University of Crete, Heraklion, Greece; ^3^Unit of Biomedical Data Analysis, Department of Mother and Child Health, University of Crete, Heraklion, Greece; ^4^Max Planck Institute of Biophysics, Frankfurt, Germany

**Keywords:** *Coxiella burnetii*, serodiagnostic markers, ELISA, western blot, Q fever

## Abstract

*Coxiella burnetii* is the agent that causes acute and chronic Q fever infections in humans. Although the isolates studied so far have shown that the two forms of the disease differ in virulence potential thus, implying a variance in their proteomic profile, the methods used do not deliver enough discriminatory capability and often, human infections may be mis-diagnosed. The current study adds further knowledge to the results that we have already published on the Coxiella outer membrane protein 1 (Com1). Herein we identified the proteins GroEL, Ybgf, OmpH, and UPF0422 as candidates for serodiagnostics of Q fever; following cloning, expression and purification they were further used as antigens in ELISA for the screening of patients' sera associated with chronic Q fever endocarditis, sera negative for phase I IgG, sera with at least one sample positive for phase I IgG and sera from patients who suffered from various rheumatic diseases. Blood donors were used as the controls. Sensitivity, specificity, positive predictive value, negative predictive value, and Cohen's kappa coefficient (κ) were calculated and we also performed binary logistic regression analysis to identify combinations of proteins with increased diagnostic yield. We found that proteins GroEL and Ybgf, together with Com1, play the most significant role in the correct diagnosis of chronic Q fever. Of these three proteins, it was shown that Com1 and GroEL present the highest sensitivity and specificity altogether. The results add to the existing knowledge that an antigen-based serodiagnostic test that will be able to correctly diagnose chronic Q fever may not be far from reality.

## Introduction

*Coxiella burnetii*, the aetiological agent of Q fever, is a small intracellular Gram-negative bacterial pathogen that since 2009 has been shown that may survive outside an intracellular nature of parasitism (Omsland et al., 2009). The pathogen displays antigenic variations which are related to the mutational variation in its lipopolysaccharide (LPS) (Maurin and Raoult, 1999). Phase II bacteria (non-infectious) correspond to rough LPS of the Enterobacteriaceae family bacteria and are cultured *in vitro* after serial passages in cell cultures. Bacteria in phase I stage (natural phase corresponding to smooth LPS), are detected in humans and animals and are characterized of high infectivity (Maurin and Raoult, 1999). The genetic foundation of antigenic variation of *C. burnetii* was recently described by Beare et al. (2018).

Infection by the bacterium is ensued mainly by inhalation from the animal or human host. The characteristics of *C. burnetii* such as environmental stability, extremely low infectious dose (Moos and Hackstadt, 1987; Hackstadt, 1996) and aerosol route of exposure have classified the bacterium as a category B agent for bioterrorism (Oyston and Davies, 2011).

Due to the route of *C. burnetii* infection, Q fever is first presented as an acute, pulmonary disease characterized by prolonged high fever, flu-like symptoms, and pneumonia (Eldin et al., 2017). Due to these non-specific symptoms and the fact that a healthy immune system can battle against acute Q fever, the disease is largely mis- and under-diagnosed. Further contribution to under diagnosis of acute Q fever is the fact that healthy individuals can recover from acute disease without medical intervention. If properly diagnosed, acute Q fever patients are treated with doxycycline for 1–2 weeks (Kersh, 2013).

*Coxiella burnetii* can establish a persistent infection through an undefined mechanism that results in chronic Q fever. The most commonly observed manifestation of chronic Q fever is endocarditis following hematogenous spread from the lungs (Aistleitner et al., 2018). Chronic Q fever can also present as chronic fatigue syndrome (Roest et al., 2013), fibrosis (Melenotte et al., 2018a), osteomyelitis (Merhej et al., 2012), and hepatitis (Gomes et al., 2014).

During the past years a hypothesis concerning the different forms of Q fever has been formed. Briefly, the hypothesis suggests that there is no chronic from of Q fever but rather a more persistent focalized infection of the bacterium—endocarditis—with the presence or not of peripheral manifestations (e.g., liver, kidney, and splenic involvement) (Million and Raoult, 2017). Even though there is an ongoing debate among Coxiellogists on the matter, all scientists agree that early diagnosis and treatment of Q fever endocarditis is critical for patients (de Lange et al., 2019). However, diagnosing Q fever endocarditis is difficult and relies upon non-specific cardiac findings, the results of serologic or molecular tests, and/or the findings on imaging studies (Melenotte et al., 2018b).

Diagnosis of the infection is mostly based on laboratory diagnostic tools due to the polymorphic clinical symptoms that the infection causes and which are not specific for Q fever. *C. burnetii*-specific indirect diagnostic tools have been mainly used for diagnosis. Currently, diagnosis based on antibody detection is most commonly used to test for the infection. Indirect immunofluorescence assay (IFA) is the reference method however, the complement fixation test (CFT) and ELISA are, also, used. As regards IFA an IgG anti-phase II antibody titer of ≥200 and an IgM anti-phase II antibody titer of ≥50 are generally considered significant for the laboratory diagnosis of acute Q fever (Maurin and Raoult, 1999). Chronic Q fever is characterized by the presence of anti-phase I antibodies, and an IgG anti-phase I antibody titer of ≥800 is generally considered to be highly predictive of Q fever endocarditis. However, the choice of cut-off titers depends on the amount of background antigen stimulation in the population under study and varies from one area to another (Maurin and Raoult, 1999; Angelakis and Raoult, 2010).

Nevertheless, IFA seems to have a number of disadvantages, such as the requirement of acute and convalescent sera, the objectivity of the interpretation of the results, potential antibody cross-reactions (giving occasionally false positive results), the need of expertized personnel, etc. ELISA is performed more easily while the interpretation is less subjective compared to IFA and automation is a choice. However, commercially available ELISA tests seem to lack the ability of IFA to identify patients who may be at risk for developing chronic Q fever (Herremans et al., 2013).

In this context, we have identified and determined antigenic proteins which could be used for the development of a chronic Q fever specific ELISA. Four *C. burnetii* proteins (GroEL, Ybgf, OmpH, and UPF0422) were chosen as potential bacterial antigens capable of differentially diagnosing chronic Q fever in humans. These proteins have already been studied at some extent in past studies described in the literature (Kowalczewska et al., 2012; Xiong et al., 2012). In particular, CBU_1718 (GroEL) and CBU_0092 (YbgF) have been detected in the blood sera of both mice and humans infected with *C. burnetii* (Xiong et al., 2012). CBU_0612 (OmpH) is required for the release of the translocated proteins from the plasma membrane (Dumetz et al., 2006), while CBU_0937 (UPF0422) has been described as an antigenic target for the diagnosis of Q fever (Sekeyova et al., 2010).

The results of the current work together with our results of the Com1 (Vranakis et al., 2019) may be considered as a first step for the production of a serodiagnostic test that will be able to correctly diagnose chronic Q fever.

## Materials and Methods

### Bacteria Strains, Oligonucleotides and Media

*Escherichia coli* strain DH5α and BL21 (DE3) were used as a host for cloning and expression of recombinant proteins, respectively. *Escherichia coli* cells were grown in lysogeny broth (LB) medium at 37°C. Synthetic oligonucleotides, obtained from Eurofins Genomics, are listed in Supplementary Table 1.

### Construction of the Expression Vectors

Genomic DNA from *Coxiella burnetii* was isolated using the Qiamp Tissue kit (Qiagen, Hilden, Germany) according to the manufacturer's specifications. Primers were designed based on the genome sequence of *C. burnetii* RSA 493/Nine Mile phase I (NCBI: NC_002971). Four full-length genes (CBU_1718, CBU_0092, CBU_0937 and CBU_0612) were amplified by PCR using Phusion DNA polymerase (Thermo Fisher Scientific). The PCR amplified products were purified from the agarose gel using Zymoclean Gel DNA Recovery kit (Zymo Research) and then digested with NdeI and XhoI. Subsequently, the digested fragments were ligated into the corresponding sites of pET-22b(+) to generate the expression vectors. In addition, for the target CBU_0937, two primers (CBU_0937_pelB_Fw and CBU_0937_pelB_Rev) was used to amplify the region encoding the mature protein without the first 23 amino acids according to the predicted signal peptide cleavage site. The resulting DNA fragment was cloned into the NcoI and XhoI sites of the pET-22b(+) vector using the InFusion ligation-independent cloning method (Takara Bio). All final constructs were verified by DNA sequencing (Eurofins Genomics) (Supplementary Figure 1).

### Expression and Purification of Recombinant Proteins

The expression vectors were transformed into *E. coli* BL21 (DE3) cells. After selection on agar plates, a single transformant was used to inoculate 50 ml of LB medium containing 50 μg/ml carbenicillin and incubated at 37°C overnight. Twenty five milliliter of this pre-culture was used to inoculate 1 l LB medium supplemented with 50 μg/ml carbenicillin. The culture was incubated at 37°C and 180 rpm until the optical density (OD) at 600 nm reached 0.5–0.7. The protein production was induced by addition of 1 mM isopropyl β-D-1-thiogalactopyranoside (IPTG) and incubation was continued at 37°C for 4 h. Cells were harvested by centrifugation at 4,300 ×g for 10 min.

Cells were resuspended in the lysis buffer [20 mM HEPES, 300 mM NaCl, 1 mM EDTA, 1 mM phenylmethanesulfonyl fluoride (PMSF), 20 μg/ml DNase I, pH 7.5] at a ratio of 1 g wet cell per 5 ml buffer, and incubated on ice for 20 min. Cells were disrupted by passing through a French pressure cell at 19,000 psi (SLM Aminco FA-078). After centrifugation at 17,400 ×g and 4°C for 20 min to remove unbroken cells and cell debris, membranes and soluble fractions were separated by centrifugation at 200,000 ×g and 4°C for 1 h.

The His-tagged recombinant proteins were purified by Ni-NTA affinity chromatography. For two soluble proteins (CBU_1718 and CBU_0092), soluble fractions obtained from the previous ultracentrifugation step were loaded onto a 4 ml Nickel-NTA column (IBA), which was pre-equilibrated with 300 mM NaCl, 20 mM HEPES, and 10 mM imidazole, pH = 7.5. After loading the sample, two washing steps of 10 and 30 mM of imidazole, respectively, were performed. The target proteins were eluted with five column volumes of binding buffer (300 mM NaCl and 20 mM HEPES, pH = 7.5) supplemented with 300 mM imidazole. For two membrane proteins (CBU_0937 and CBU_0612), membranes were resuspended in 20 mM HEPES, 300 mM NaCl, 1 mM EDTA, 1 mM phenylmethanesulfonyl fluoride (PMSF), pH = 7.5 to a final concentration of 5 mg/ml and were solubilized in the presence of 1% (w/v) n-Dodecyl β-D-maltoside (DDM) at 4°C for 1 h. The insoluble membrane fraction was removed by ultracentrifugation at 200,000 ×g and 4°C for 1 h. The supernatant containing solubilized membrane proteins were collected and the membrane proteins were purified by Ni-NTA column, as described above, except that all buffers contained 0,02% DDM. The eluted proteins were concentrated using a 30 kDa cut-off Amicon concentrator (Merck Millipore) to a final volume of 1 ml. Protein concentration was determined using the BCA assay (Thermo Scientific Pierce).

### SDS-PAGE and Western Blot

Sodium dodecyl sulfate-polyacrylamide gel electrophoresis (SDS-PAGE) was performed using 12% acrylamide gel followed by Coomassie blue staining. The His-tagged recombinant proteins were immunodetected using a monoclonal anti-poly-histidine alkaline phosphatase conjugated antibody (Sigma) following the manufacturer's instructions. The color reaction was developed with 5-bromo-4-chloro-3-indolyl phosphate (BCIP) and nitroblue tetrazolium (NBT). In addition, sera from patients diagnosed with acute or chronic Q fever were used to evaluate the ability of individual recombinant proteins to identify the chronic Q fever. AP-conjugated anti- human IgG was used as a secondary antibody.

### Serum Samples

Sample sera used herein were retrieved from the collection of sera samples kept at the Hellenic national reference center for Q fever (Crete, Greece). All patients were asked to fill in a consent form that allowed future research studies to be performed by using their samples. Sample sera were initially tested with IFA for IgG and IgM antibodies against phase II *C. burnetii*, using a commercially available antigen (*C. burnetii* spot IF; BioMérieux). Based on past seroprevalence studies carried out by our laboratory in Crete, titers of ≥1/1,920 for IgG or 1/480 for IgG together with 1/200 for IgM were considered as cut-offs that would describe positive samples. All sera which were received from patients under the suspicion of chronic Q fever (endocarditis, hepatitis, etc.), were, also, tested for phase I IgG antibodies, using a commercially available IFA kit (FOCUS Diagnostics). The cut-off of ≥1/1,024 was considered as positive for chronic Q fever according to the accepted Duke's criteria (Wegdam-Blans et al., 2012). None of the samples was tested by PCR for *C. burnetii*.

Apart from the above samples, sera drawn from blood donors (collected during a previous study carried out at the laboratory) (Vranakis et al., 2012) and sera collected from patients with chronic inflammatory rheumatic diseases (Systemic Lupus Erythromatosus, Rheumatoid Arthritis), were used as disease control sera.

All sera that were chosen for analyses using the under-development ELISA method were re-tested using the above-mentioned FOCUS diagnostic kit.

### ELISA Development and Optimization

Checkerboard titrations were performed by indirect ELISA method in order to identify the optimum antigen, and conjugate concentration as described elsewhere (Crowther, 2000). Microtiter flat bottom plates (96 wells, Corning Costar) were coated with different concentrations of purified recombenant proteins (1.0, 0.5, 0.25, 0.125, 0.0625, 0.03, and 0.015 μg/ml) diluted in carbonate-bicarbonate buffer (pH 9.6) and incubated at 4°C overnight. The wells were then washed with PBS-T washing buffer (phosphate-buffered saline + 0.05% [v/v] Tween 20) and blocked with blocking buffer (PBS-T with 1% [w/v] bovine serum albumin [BSA]) at 37°C for 60 min. After blocking, the wells were incubated with a solution of Q fever positive serum (1:25–1:6,400 serially diluted in blocking buffer) for 60 min at 37°C, washed three times with washing buffer, and incubated with peroxidase conjugated rabbit anti-human IgG (1:3,000 diluted in blocking buffer, Boster Biological Technology) at 37°C for 1 h. Following three washing cycles, the wells were incubated with a solution of substrate/chromogen (TMB Core Biorad) for 10 min and the colorimetric reaction was stopped by adding 0.5 M H_2_SO_4_. All reagents were added at a volume of 100 μl/well. The optical density was read at 490 nm on a microplate reader (Multiskan FC, Thermo Fisher Scientific).

The optimal concentration for the antigen was determined by the lowest concentration which demonstrated the positivity of the reaction at any dilution of the positive serum. The optimal dilution of the serum was presented by the greatest difference in reading between the positive and negative serum in the optimal antigen concentration.

### Statistical Analysis

To perform the statistical analysis (IBM SPSS Statistics v.25), *p* < 0.05 were set as statistically significant. For each protein, a receiver operating characteristic (ROC) curve analysis was built; following the area under curve (AUC) estimation, the cut-off value maximizing the Youden's index (J=sensitivity+specificity-1) was selected as optimal cut-off value. Each protein was considered as positive for the new ELISA after been classified into the two categories: 0 = “no disease” or 1 = “disease,” by considering each value < cut-off as 0 (no disease) and each value ≥ cut-off as 1 (disease). Following the above steps, a classification 2 × 2 table of each protein was obtained in reference to the standard clinical classification. The values of true positives, true negatives, false positives, and false negatives of that table were used to calculate a series of indicators of the agreement between the classification obtained by the protein and the existing clinical classification, that is indicators of the performance of the protein as a possible diagnostic test. In particular, we calculated the sensitivity, specificity, positive predictive value, negative predictive value, and Cohen's kappa coefficient (κ) for each protein.

Except from the above approach we, also, processed the protein values using Binary logistic regression analysis to find any possible combinations of proteins that could act as better diagnostic factors.

## Results

### Expression and Purification of the Recombinant Proteins

Continuing our earlier work (Vranakis et al., 2019), in the current study four proteins were selected for validation of their antigenic properties with respect to their capacity to differentiate the chronic from acute infections of Q fever. Among them, two (CBU_0937 and CBU_0612) are predicted as outer membrane proteins, and two (CBU_1718 and CBU_0092) are predicted to be localized in the periplasm of *C. burnetii* (Supplementary Table 2). For the heterologous expression of those antigenic proteins in *E. coli*, full-length genes were amplified from *C. burnetii* genomic DNA and subsequently cloned into the NdeI/XhoI sites of pET-22b(+), resulting in expression vector with C-terminal His_6_-tag and N-terminal native signal peptide, if any is present (Supplementary Figure 1). The expression vectors were transformed into *E. coli* BL21 (DE3) and small-scale expression trials were conducted by varying growth temperatures, induction time and concentrations of inducers (data not shown). It was shown that under the conditions tested, no apparent expression can be observed for CBU_0937 (data not shown), while the expression of the other three proteins was detected by immunodetection. In order to obtain the recombinant protein of CBU_0937, its predicted signal sequence was removed and the region encoding the mature protein was fused to the *pelB* leader sequence. By employing the *pelB* leader sequence for periplasmic localization in *E. coli*, significant expression of CBU_0937 was achieved (Supplementary Figure 2).

**Figure 1:**
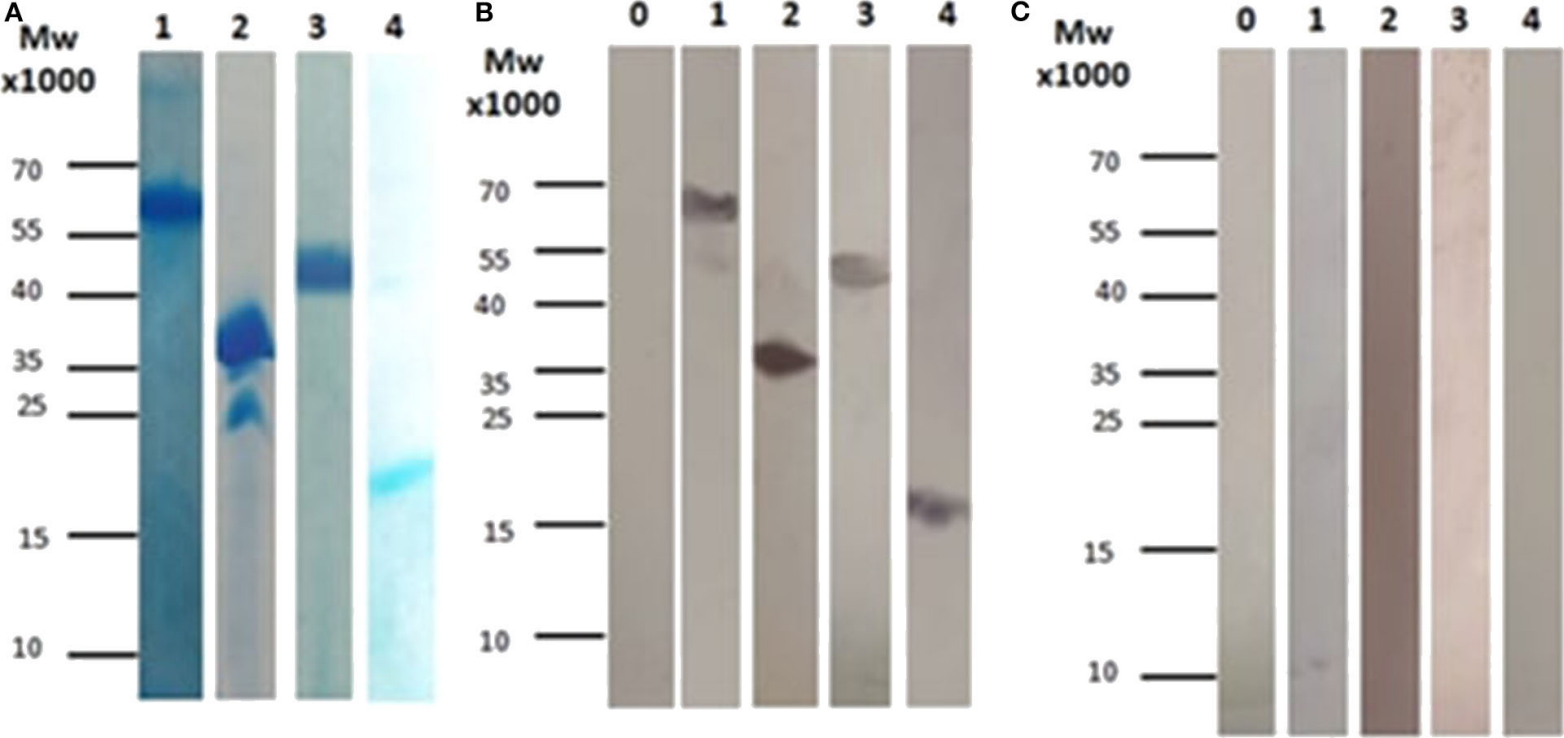
**(A)** SDS-PAGE for all the subunits after affinity chromatography purification. (1) Corresponds to CBU_1718, (2) Corresponds to CBU_0092, (3) Corresponds to CBU_0937, and (4) Corresponds to CBU_0612. **(B)** Immunoblotting using sera from patients with chronic Q fever. (1) Corresponds to CBU_1718, (2) Corresponds to CBU_0092, (3) Corresponds to CBU_0937, (4) Corresponds to CBU_0612 and 0. Corresponds to whole cell lysate before induction (used as control). **(C)** Immunoblotting using sera from patients with acute Q fever. (1) Corresponds to CBU_1718, (2) Corresponds to CBU_0092, (3) Corresponds to CBU_0937, (4) Corresponds to CBU_0612 and 0. Corresponds to whole cell lysate before induction (used as control).

For all four proteins, inducer concentration of 1 mM IPTG was found to lead to high production levels. Four hours after induction, cells were harvested and the antigenic proteins of interest were purified using Ni-NTA chromatography. The Coomassie-stained SDS-PAGE gels demonstrated that all four proteins can be purified to relatively high purity in one affinity purification step (Figure 1A). In addition, the apparent molecular masses observed for the purified proteins in SDS-PAGE matched the calculated masses of the respective antigenic proteins.

### Testing of Sera of Patients With Acute and Chronic Q Fever by Western Blot

All purified recombinant proteins were tested for their ability to diagnose acute or chronic Q fever. For this aim, serum samples from patients diagnosed with acute or chronic Q fever were used in immunoblotting. Three serum samples from patients suffering from the chronic or the acute form of the disease were tested individually in a total of six different western blots. Those sera were later used among others in the ELISA assay. Our results showed that all proteins can be detected in patients suffering from chronic Q fever (Figure 1B), while no immunoreaction was observed when the serum from patients with acute Q fever was used (Figure 1C).

### Testing of Sera by IFA

A total of 160 serum samples were re-tested using the IFA assay. Testing of the sample sera was carried out as previously described (Vranakis et al., 2019). Table 1 summarizes the sera samples of the patients that were tested again by IFA and the indirect ELISA that we developed. All 20 samples that were considered as belonging to chronic patients were tested positive by IFA for phase I *C. burnetii*. Titers ranged from 1/1,024 to 1/65,536. Accordingly, all 61 sample sera corresponding to acute Q fever were tested positive by IFA; of these samples, 46 (75.4%) revealed titers against IgG only, while the rest 15 (24.6%) revealed titers against both IgM (titers ranging from 1/200 to 1/1,600) and IgG (titers ranging from 1/480 to 1/7,680). Of the blood donors, surprisingly one revealed antibodies against IgG phase I (titer 1/1,024). Unfortunately, we could not trace this blood donor and collect more data. Our best guess is that he had come into contact with the pathogen at some time however, he never developed any symptoms or had the disease and was not aware of it. Of the 47 samples that corresponded to acute Q fever, 32 (68.1%) showed antibodies against phase I IgG, also, with the titers ranging from 1/1,024 to 1/4,096. Of the 20 samples that corresponded to patients suffering from any kind of rheumatoid disease, at least half of them [55% (11/20)] revealed antibodies against phase II IgG with titers ranging from 1/1,920 to 1/7,680, while three [15% (3/20)] revealed antibodies against phase I IgG with titers ranging from 1/1,024 to 1/8,192.

**Table 1:** **(A)** Sample sera categorized based on clinical diagnosis were tested by IFA for acute Q fever. Samples were considered positive for acute Q fever by IFA if they met the criteria of IgG ≥ 1/1,920 or IgG ≥ 1/480 and IgM 1/200. **(B)** Distribution of the 160 samples tested based either on clinical diagnosis or on the IFA result for IgG phase I against *C. burnetii*.

	**Number of sample sera**	**Clinical diagnosis**	**Laboratory diagnosis**	
**A**			**Positive for acute Q fever**	**Positive for chronic Q fever**
	20	Chronic Q fever		100%
	12	Blood donors		8.3% (1/12)
	61	Acute Q fever	100%	0
	47	Acute Q fever (at least one sample of each	100%	68.1% (32/47)
		patient was tested ≥1/1,024 IgG phase I)		
	20	Rheumatoid disease	55% (11/20)	15% (3/20)
**B**	**Result (for chronic Q fever)**	**Clinical diagnosis**	**Laboratory diagnosis**	
	Negative	132	103	
	Positive	28	56	
	**Total**	160	160	

*The cut-off for a sample to be considered as positive for chronic Q fever by IFA was ≥ 1/1,024*.

### Indirect ELISA

At first we used the results that we had retrieved from a similar study that we performed on CBU_1910 (Vranakis et al., 2019). Based on that study, the titration of CBU_1910 and Q fever positive sera determined by indirect ELISA showed high levels of reactivity in nearly all combinations of concentrations tested. Based on these results, the ideal protein concentration that would serve as antigen in the indirect ELISA that could give us the highest reactivity and the minimum “background” interference was defined at 1 μg/l with a serum dilution of 1/100. All four proteins were tested in separate and ended up at similar results.

Of the 20 samples corresponding to chronic cases, all samples revealed ODs above the cut-off for the protein CBU_1718, while only one (that was strongly positive by IFA) failed to overpass the cut-off of proteins CBU_0092, CBU_0612 and CBU_0937. Of the 12 blood donors' samples, proteins CBU_0092 and CBU_0612 gave negative results agreeing with the IFA, while one sample was over the cut-off for protein 1718 and four samples were over the cut-off for protein 0937. Of the 20 samples corresponding to the patients suffering from rheumatoid disease, none overpassed the cut-off for CBU_0092, while six (6, of which two agreed with IFA) were over the cut-off for protein CBU_1718, 11 (of which three agreed with IFA) for protein 0612 and 14 (of which three agreed with IFA) for protein CBU_0937. As regards the 61 samples that corresponded to the patients designated as negative for chronic Q fever, nine (9) for protein CBU_1718 and eight (8) for protein CBU_0937, while none was positive for proteins CBU_0092 and CBU_0612. Lastly, regarding the 47 samples with an at least one titer ≥1/1,024 by IFA, seven (7) were positive for CBU_1718, one (1) for proteins CBU_0092 and CBU_0612 and 12 for protein CBU_0937.

### Statistical Analysis

The patients tested were initially divided into five groups; (a) chronic patients (based both on laboratory and clinical examination), (b) healthy blood donors, (c) patients whose samples were negative for phase I IgG, (d) patients whose at least one sample was positive for phase I IgG, and (e) patients suffering from various rheumatic diseases (rheumatoid arthritis, systemic lupus erythematosus). During the second step of the analysis, samples were divided into two categories, those (28/160) presenting an IgG phase I titer of ≥1/1,024 and those (132/160) below this cut-off point.

**Figure 2:**
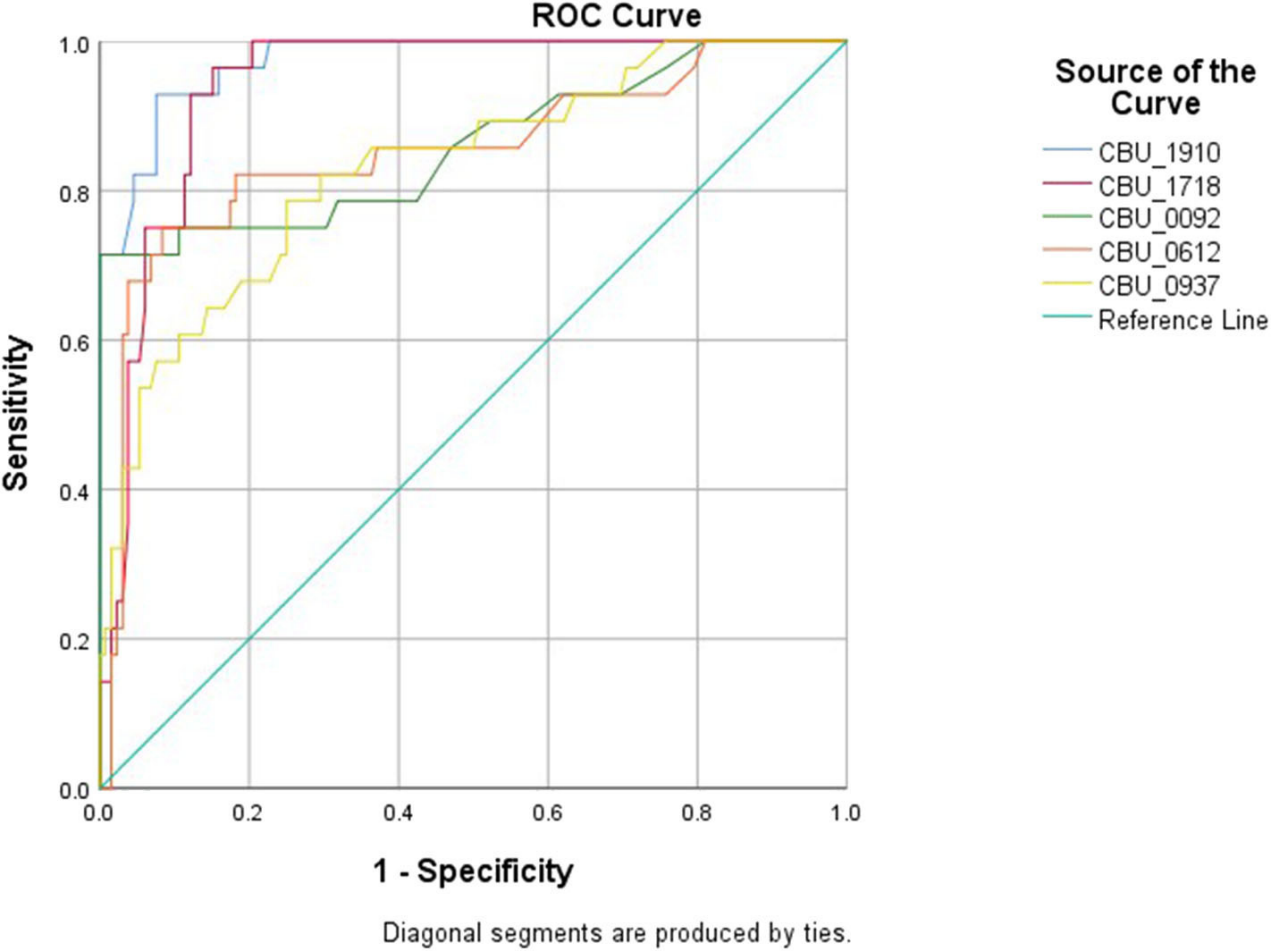
Specificity and sensitivity of each protein based on the ROC analysis.

**Figure 3:**
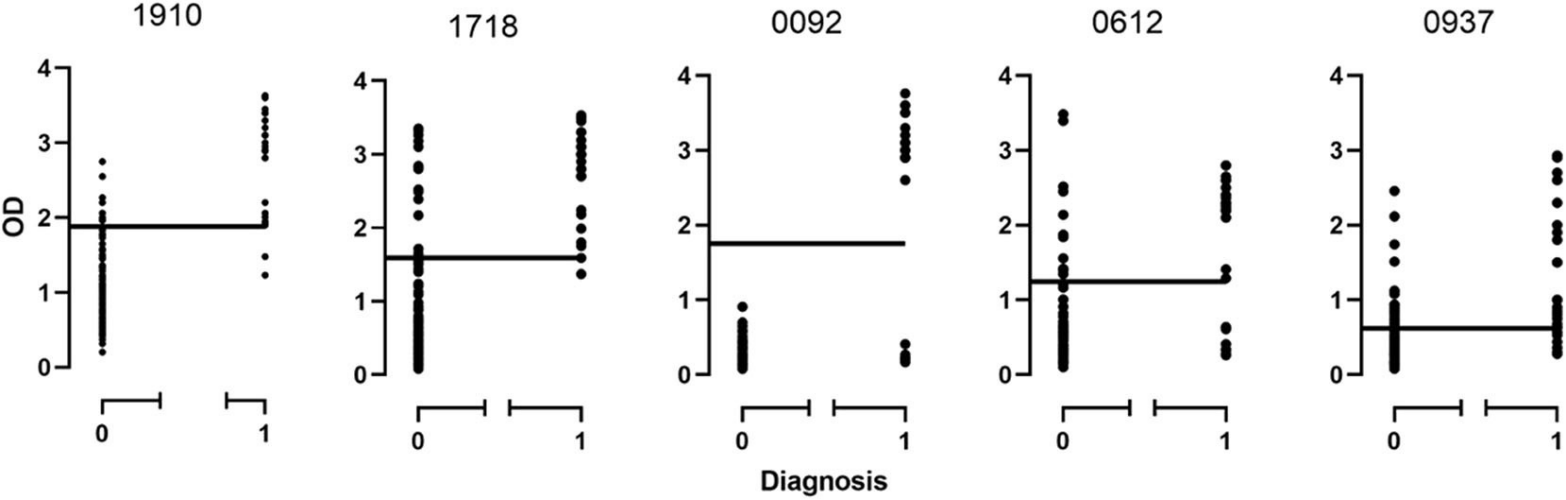
Distribution of ODs for each protein based on the disease state. 0, non-chronic Q fever; 1, chronic Q fever.

The cut-off point for each protein was calculated based on the above mentioned cut-off according to which, samples were assigned as originating from chronic patients (Table 1, Figure 2). All proteins seem to produce statistical results, while CBU_1910 seem to be the most sensitive one of the five proteins tested. On the other hand, CBU_0937 seemed to have the least sensitivity but together with CBU_0092 they seemed to produce the most specific results. Based on the proteins' cut-off we calculated the discrepancy between the result obtained following the final diagnosis set up by the clinicians against that of the ELISA testing (Table 2, Figure 3). Based on the OD values calculated, CBU_1910 seems to have a reasonable sensitivity and specificity and so is the case for CBU_0092. The rest three proteins (CBU_1718, CBU_0612, CBU_0937) seem to have a high sensitivity but they seem to lack specificity although CBU_1718 did not fail to detect almost no true positive sample.

**Table 2:** True and False Negative, True and False Positive values as calculated based on the ROC curve analysis for each protein in separate.

**Protein**	**Clinical diagnosis**	**ELISA**		
		**Negative**	**Positive**	**Total**
1910	Negative	122	10	132 (82.5%)
	Positive	2	26	28 (17.5%)
	Total	124 (77.5%)	36 (22.5%)	160
1718	Negative	112	20	132 (82.5%)
	Positive	1	27	28 (17.5%)
	Total	113 (70.6%)	47 (29.4%)	160
0092	Negative	132	0	132 (82.5%)
	Positive	8	20	28 (17.5%)
	Total	140 (87.5%)	20 (12.5%)	160
0612	Negative	121	11	132 (82.5%)
	Positive	7	21	28 (17.5%)
	Total	128 (80%)	32 (20%)	160
0937	Negative	99	33	132 (82.5%)
	Positive	6	22	28 (17.5%)
	Total	105 (65.6%)	55 (34.6%)	160

*The number of samples agreeing with the clinical diagnosis and presenting with proteins above or below the cut-off set up for each one in the current study can be extracted*.

Of the 20 samples corresponding to chronic Q fever, proteins CBU_1910 and CBU_1718 were positive for all those, while proteins CBU_0092, CBU_0612, and CBU_0937 were positive for 19 of them. Of the rest of the samples that corresponded to patients suffering from other forms of the disease or other conditions or were even healthy, six (6/140; 4.3%) were positive for CBU_1910, 19 (13.6%) were positive for CBU_1718, 11 (7.8%) were positive for CBU_0612, 26 (18.6%) were positive for CBU_0937, while none was positive for CBU_0092.

In the case of protein CBU_1910, 92.4% (122/132) of the samples agreed with the negative clinical diagnosis and 92.8% (26/28) of the samples agreed with the positive clinical diagnosis. Similar results were found for the rest of the proteins. Only protein CBU_0092 presents with a 100% agreement with clinical diagnosis demonstrating its high specificity.

The ROC curve analysis was then performed (Supplementary Table 3) and the true and false negative and the true and false positive values were calculated for each protein (Supplementary Table 4). Furthermore, for all proteins, we have calculated a significant correlation to the presence of the disease (all *p*-values were 0.00) while the area under the curve ranged from 0.83 (CBU_0937) to 0.942 (CBU_1718). Several other factors were also calculated including true positive and negative rates, predictive values, likelihood ratios, the probability of agreement by chance and the κ coefficient (Supplementary Table 4). It has been showed from the previous work (Vranakis et al., 2019) and now from the current study that proteins CBU_1910, CBU_1718, and CBU_0092 play the most significant role in the correct diagnosis of chronic Q fever. Of these three proteins, CBU_1910 and CBU_1718 present the highest combined sensitivity and specificity; proteins CBU_0092 and CBU_0612 do present very high specificities (close to 100%) but they do not seem to be sensitive enough which may lead to false negative results.

Apart from the ROC curve analysis we, also, tried to process the proteins following the binary regression analysis in order to search for possible combinations of the proteins that could increase their diagnostic value. For this purpose, all the above factors were again calculated (Supplementary Tables 5, 6) for each individual protein (CBU_1910, CBU_1718, CBU_0092, CBU_0937, CBU_0612) and for any possible protein combinations. When recalculating the results of protein CBU_1910 for example it can be seen that using the binary regression analysis, 97% (128/132) instead of 92.4% of the samples tested agreed with the negative clinical diagnosis and 71.4% (20/28) instead of 72.2% of the samples tested agreed with the positive clinical diagnosis. However, it seems that whatever combination is used, modest changes are achieved in terms of true and false positive rates, compared to the values obtained for each protein alone (Supplementary Table 6). Three more factors (B, exponential B and constant) were further calculated in an attempt to build up a model (Supplementary Table 6). This attempt was not successful since whatever combination we tried the model kept working with one of the proteins used keeping the rest one(s) constant. And again, proteins CBU_1910, CBU_1718 and to a lesser extend CBU_0092 seem to play the most important role in the correct diagnosis of the disease. In our case, the Exp(B) demonstrates the possibility for a person to suffer from the disease if his sample is detected over the cut-off for each protein compared to a person whose sample is tested below the cut-off set in the present for the same protein.

## Discussion

The major goal of the current study was the development of a reliable tool for the differential diagnosis of Q fever based on antibody detection. Five proteins, four studied herein (CBU_1718, CBU_0092, CBU_0612, and CBU_0937) and one (CBU_1910) studied in an earlier study of ours were chosen as potential diagnostic markers. These proteins had been described in an earlier study by our team (Papadioti et al., 2011), and gained consideration according to further studies described in the literature (Sekeyova et al., 2010; Kowalczewska et al., 2012; Xiong et al., 2012; Wang et al., 2013; Skultety, 2017).

According to Xiong et al. (2012), CBU_1718 (GroEL) and CBU_0092 (YbgF) were recognized in the blood sera of both mice and humans infected with *C. burnetii*. In addition, these two proteins have been previously described as major seroreactive antigens (Chao et al., 2005; Coleman et al., 2007; Sekeyova et al., 2009). On the other hand, CBU_0612 (OmpH) is an 18-kDa outer membrane chaperon protein that is required for the release of the translocated proteins from the plasma membrane (Dumetz et al., 2006). This protein has been documented as a very strong immunodominant marker for both acute and chronic forms of the Q fever disease (Kowalczewska et al., 2012). CBU_0937 (UPF0422) was first described as an antigenic target for the diagnosis of Q fever by Sekeyova et al. (2010). According to their study, this protein appears to have low value of sensitivity but high range of specificity against Q fever, which could lead to false negative results. Nevertheless, the authors suggest that CBU_0937 may be used together with CBU_1910 as a diagnostic tool.

In adding to the mentioned above, Skultety (2017) described in their 2017 review that CBU_1718 (GroEL) appears to be a very reliable molecular marker for serodiagnosis of both acute and chronic Q fever and CBU_1910 (Com1) acts as an antigen which might induce protective immunity. Furthermore, it has been suggested that CBU_0092 (YbgF) is a phase II specific marker that can be employed for the early diagnosis of acute infection. According to the same review, CBU_0612 (OmpH) is a promising candidate marker for acute and chronic Q fever.

In general, CBU_1910 (Com1) has been described in the literature as a credible diagnostic marker, exposed on the surface of *C. burnetii*. Furthermore, this protein has been perfectly described by Vranakis et al. (2019), as a very good marker for the differential diagnosis of the chronic form of Q fever. More specifically, an ELISA assay was used in their study in order to screen blood sera from patients suffering either from the chronic or the acute phase of the disease. The results of the study produced promising results as to the protein's ability to diagnose chronic Q fever with high sensitivity (92.9%) and specificity (92.4%) (Vranakis et al., 2019).

In the current study, we tested Com1, GroEL, Ybgf, OmpH, and UPF0422 for their ability to detect antibodies specific for the chronic Q fever. The results revealed that all these proteins can indeed respond to sera from patients suffering from the chronic form of the disease, while they appear to have limited to no response to sera from patients with acute Q fever. The statistical analysis suggested high sensitivity and specificity to chronic Q fever for CBU_1910 (Com1) and CBU_1718 (GroEL), while CBU_0092 (YbgF) and CBU_0612 (OmpH) appear to be specific but not sensitive enough, which may lead to false negative results. As a conclusion, Com1, GroEL, and YbgF could be considered as reliable antigenic candidates for the development of a chronic Q fever specific diagnostic tool.

Concerning the timely diagnosis of chronic Q fever, a diagnostic problem exists since the currently gold standard method for the diagnosis, immunofluorescence, has several disadvantages. To mention just a few, the need for acute and convalescent sera, the dependence of the interpretation of the results on the personnel skills, potential antibody cross-reactions that can result in false-positive results, the requirement of expertized personnel, etc. The main goal of the current study was to use the proteins mentioned above as a substitute for the development of a fast, reliable, valid and easy to use diagnostic kit, which will be based on immunochromatography. Even though there are commercially available diagnostic kits for several pathogens, such as for *Legionella* (Diederen and Peeters, 2006), *Plasmodium* (Katakai et al., 2011), and *Campylobacter* (Gomez-Camarasa et al., 2014), there is no such a kit for the differential diagnosis of the chronic Q fever in human sera. The development of such a technique would be of particular importance since it has many advantages, such as ease in use, fast diagnosis and reliable results without the necessity of experienced personnel.

Fast and reliable diagnosis, as well as, the valid differentiation between the acute and the chronic type of the disease, play a key role in the progress of the treatment. Improving the gold standard method for the diagnosis by using the results of the current study, would help health professionals choose the correct antibiotic treatment, based on the type of Q fever (acute or chronic). The differentiation is fundamental since the chronic Q fever treatment protocol requires systematic re-examinations and treatment lasting at least 18 months. On the other hand, there are many cases of patients being mistreated because of the false diagnosis of the form of the disease. Furthermore, late diagnosis of Q fever could be life threatening.

## Conclusion

It is, therefore, reasonable to conclude that the outcome of this study could be beneficial not only for the scientific knowledge but also for the public health. The study of antigenic proteins for their specificity to the acute form of Q fever would be very interesting and promising investigation as a future perspective. The development of commercial detection kit for acute Q fever, will be able to help health professionals treat the disease properly, based on the correct differential diagnosis.

## Data Availability Statement

All datasets generated for this study are included in the article/Supplementary Material.

## Author Contributions

AP designed the study, supervised the experimental procedures, and contributed on the editing of the manuscript. EM performed the experimental procedures of cloning, purifications, and Western Blotting and contributed on the editing of the manuscript. IV performed the experimental procedures of the ELISA and the IFA and contributed on the editing of the manuscript. DC contributed on the editing of the manuscript and on the statistical analysis. EY performed the statistical analysis. SK performed the evaluation of the patient sera. HX hosted EM, helped on the experimental procedures of cloning and contributed on the editing of the manuscript. GT supervised the study, set up the collaboration with HX and contributed on the editing of the manuscript. All authors contributed to the article and approved the submitted version.

## Conflict of Interest

The authors declare that the research was conducted in the absence of any commercial or financial relationships that could be construed as a potential conflict of interest.
