# Supplementary material for: In the Search of Potential Serodiagnostic Proteins to Discriminate Between Acute and Chronic Q Fever in Humans. Some Promising Outcomes
Source: Front Cell Infect Microbiol. 2020 Sep 18;10:557027. doi: 10.3389/fcimb.2020.557027 (PMC7531360; doi:10.3389/fcimb.2020.557027)
Supplement: Supplementary file 1 [file Data_Sheet_1.docx]

**Supplementary Materials**

**In the search of potential serodiagnostic proteins to discriminate between acute and chronic Q fever in humans. Some promising outcomes.**

Anna Psaroulaki^1^, Eirini Mathioudaki^2^, Iosif Vranakis^1^, Dimosthenis Chochlakis^1^, Emmanouil Yachnakis^3^, Sofia Kokkini^1^, Hao Xie^4^ and Georgios Tsiotis^2^

^1^Department of Clinical Microbiology and Microbial Pathogenesis, School of Medicine, University of Crete, Voutes – Staurakia, 71110, Heraklion, Crete, Greece

^2^Laboratory of Biochemistry, School of Science and Engineering, Department of Chemistry, University of Crete, Heraklion, Crete, Greece

^3^Unit of Biomedical data analysis, Department of Mother and Child Health, University of Crete, Heraklion, Crete, Greece

^4^Max Planck Institute of Biophysics, Max-von-Laue-Strasse 3, D-60438 Frankfurt am Main, Germany


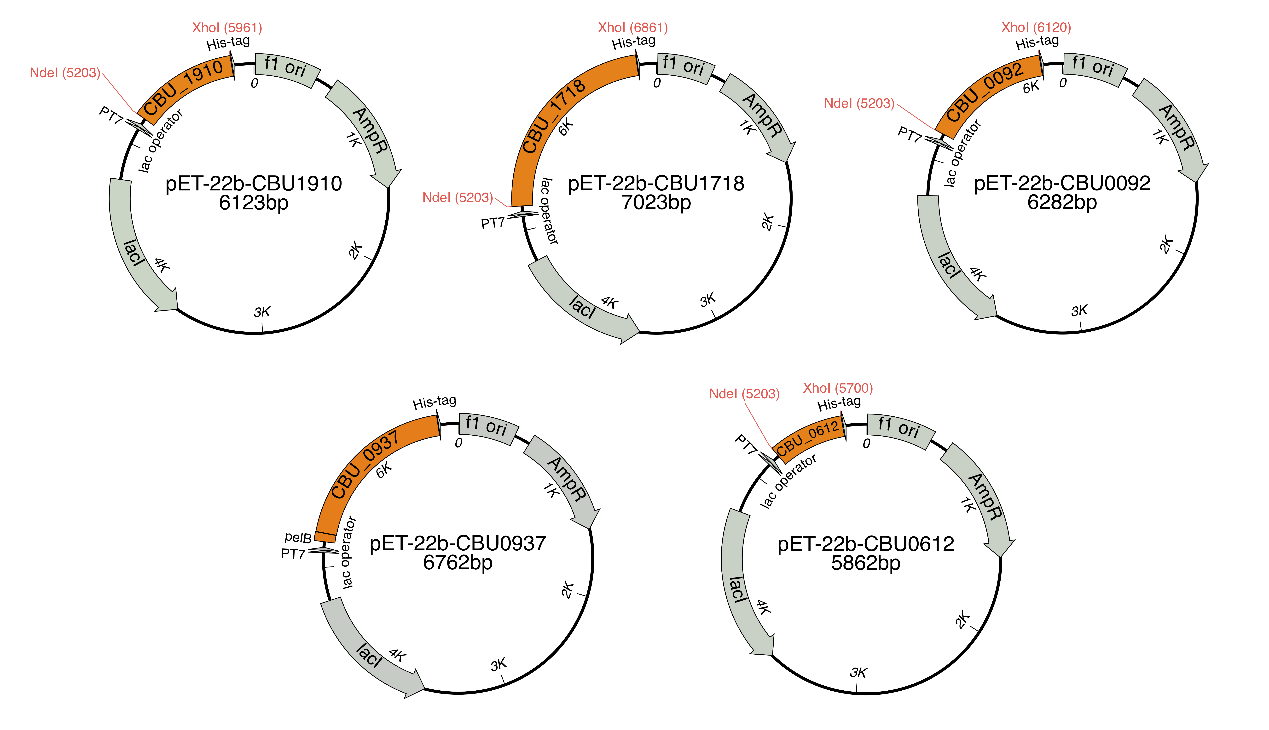


**Supplementary Figure 1:** Expression vectors used in this study. The gene encoding the protein of interest is shown in orange. NdeI and XhoI restriction sites are indicated in red. f1 ori: f1 origin of replication; AmpR: ampicillin resistance gene; lacI: lac repressor gene; PT7: T7 promoter; pelB: pectate lyase signal sequence; His-tag: C-terminal hexahistidine purification tag.


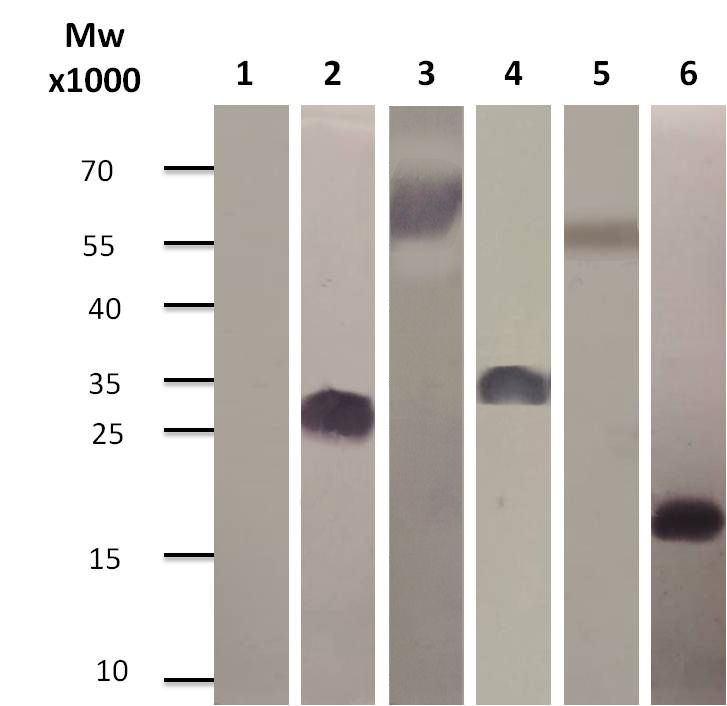


Supplementary Figure 2: Expression test for all the four different subunits. The proteins were expressed in 37^ο^C, using 1mM of IPTG. 1. Corresponds to the whole- cell lysate sample before induction (control) 2. Corresponds to CBU_1718, 3. Corresponds to CBU_0092, 4. Corresponds to CBU_0937, and 5. Corresponds to CBU_0612.

| **Oligonucleotide** | **Sequence (5’-3’)** |
| --- | --- |
| CBU_1718_Fw | GCCCATATGGCTGCAAAAGTTTTAAAATTTTCCCACGAGG |
| CBU_1718_Rev | TTACTCGAGCATCATGCCGCCCATGCCGCCCATTC |
|  |  |
| CBU_0092_Fw | GCCCATATGAGGCTTATTAAAATGAAAATAAAAACG |
| CBU_0092_Rev | TTACTCGAGAGGCGTTGTTGTTGCTGAATCGACTTC |
|  |  |
| CBU_0937_Fw | GCCCATATGACGTCCAAGCTGGTCATTTCCGCGTTGGGC |
| CBU_0937_Rev | TTACTCGAGAAAATAAAGATCGAACTGTGCCGTTACCAC |
|  |  |
| CBU_0612_Fw | GCCCATATGATTAAACGCTTGTTATCTGCTATCTG |
| CBU_0612_Rev | TTACTCGAGTTTCAATGCTGATACAACGTTTGAAG |
|  |  |
| CBU_0937_pelB_Fw | CCGGCGATGGCCATGAGTACCCCCGCTACCACTAATC |
| CBU_0937_pelB_Rev | GGTGGTGGTGCTCGAGAAAATAAAGATCGAACTGTGC |

**Supplementary Table 1:** Oligonucleotides used in the study. NdeI and XhoI restriction enzyme sites are shown in lower case.

| **Protein properties and features** | | | | **ELISA results** |
| --- | --- | --- | --- | --- |
| **Gene** | **Gene ID/Protein** | **Mw (KDa)** | **Predicted localization** | **Cut-off** |
| *com1* | CBU_1910 | 27.6 | Outer Membrane | 1.88 |
| *groL* | CBU_1718 | 58.3 | Periplasm | 1.5895 |
| *ybgF* | CBU_0092 | 34.2 | Periplasm | 1.755 |
| *upf0422* | CBU_0937 | 51.4 | Outer Membrane | 1.2465 |
| *ompH* | CBU_0612 | 18.8 | Outer membrane | 0.62 |

**Supplementary Table 2:** The antigenic proteins of interest and their features are demonstrated. Furthermore, the cut-offs calculated for each protein following the interpretation of the ELISA values against the IFA titres are also shown.

| **Area under the curve** | | | | | |  |  |  |  |  |
| --- | --- | --- | --- | --- | --- | --- | --- | --- | --- | --- |
| **Proteins** | Area | Std. error | Asymptotic Sig. | Asymptotic 95% confidence interval | |  |  |  |  |  |
|  |  |  |  | Lower bound | Upper bound | **Sensitivity** | **Specificity** | **PPV (%)** | **NPV (%)** | **Kappa value (κ)** |
| CBU_1910 | 0,974 | 0,012 | 0,000 | 0,951 | 0,997 | 0,929 | 0,924 | 92,85 | 92,42 | 0,767 |
| CBU_1718 | 0,942 | 0,018 | 0,000 | 0,907 | 0,976 | 0,964 | 0,848 | 96,42 | 84,84 | 0,641 |
| CBU_0092 | 0,860 | 0,048 | 0,000 | 0,767 | 0,954 | 0,714 | 1,000 | 71,42 | 100 | 0,805 |
| CBU_0612 | 0,852 | 0,047 | 0,000 | 0,760 | 0,944 | 0,750 | 0,917 | 75 | 91,66 | 0,631 |
| CBU_0937 | 0,830 | 0,044 | 0,000 | 0,743 | 0,917 | 0,786 | 0,750 | 78,57 | 75 | 0,388 |

**Supplementary Table 3:** Values calculated for each protein based on the Area Under the Curve analysis. Significance was set at > 0.05. The test performance indicators calculated for each protein based on the comparison between the ELISA and the IFA testing are also shown. PPV: Positive Predictive Value. NPV: Negative Predictive Value.

| **Protein** | **TPR** | **TNR** | **PPV** | **NPV** | **FPR** | **FNR** | **lr+** | **lr-** | **OR** | **Acc** | **95% CI** | **PE** | **κ** |
| --- | --- | --- | --- | --- | --- | --- | --- | --- | --- | --- | --- | --- | --- |
| 1910 | 0,929 | 0,924 | 0,722 | 0,984 | 0,076 | 0,071 | 12,257 | 0,077 | 158,6 | 0,925 | 32.8-767 | 0,679 | 0,767 |
| 1718 | 0,964 | 0,848 | 0,574 | 0,991 | 0,152 | 0,036 | 6,364 | 0,042 | 151,2 | 0,869 | 19.4-1176.7 | 0,634 | 0,641 |
| 0092 | 0,714 | 1,000 | 1,000 | 0,943 | 0,000 | 0,286 | - | 0,286 | - | 0,950 | - | 0,744 | 0,805 |
| 0612 | 0,750 | 0,917 | 0,656 | 0,945 | 0,083 | 0,250 | 9 | 0,273 | 33 | 0,888 | 11.5-94.8 | 0,695 | 0,631 |
| 0937 | 0,786 | 0,750 | 0,400 | 0,943 | 0,250 | 0,214 | 3,143 | 0,286 | 11 | 0,756 | 4.1-29.5 | 0,602 | 0,388 |

**Supplementary Table 4:** Statistical factors associated with the comparison between the indirect immunofluorescence antibody test (IFAT) and the recombinant protein-based ELISA for the detection of serum phase I IgG antibodies against *C. burnetii*. The statistical analysis was performed based on the ROC. TPR: True Positive Rate = sensitivity = probability of detection = power = tpr = tp/(tp+fn). TNR: True Negative Rate = specificity = selectivity = true negative rate = tnr = tn/(tn+fp). PPV: Positive Predictive Value = 1-specificity = precision = confidence = tp/(tp+fp). NPV: Negative Predictive Value = tn/(tn+fn). FPR: false positive rate = probability of false alarm, fpr= fp/(fp+tn). FNR: false negative rate = miss rate = fnr = fn/(fn+tp). lr+: positive likelihood ratio = lr+ = tpr/fpr. lr-: negative likelihood ratio = lr- = fnr/tnr. OR: Odds ratio = lr+/lr-. Acc: accuracy = classification rate, acc = (tp+tn)/(tp+tn+fp+fn). 95% CI= 95% Confidence Interval. PE: the probability of agreement by chance, pe = [(tp+fp)x(tp+fn)+(tn+fp)x(tn+fn)]/[(tp+tn+fp+fn)**2]. κ: Cohen's kappa coefficient of agreement, κ = (acc-pe)/(1-pe).

| **Protein or combinations** | **Clinical diagnosis** | **ELISA** | |  |
| --- | --- | --- | --- | --- |
|  |  | Negative | Positive | Total |
| 1910 | Negative | 128 | 4 | 132 (82.5%) |
|  | Positive | 8 | 20 | 28 (17.5%) |
|  | Total | 136 (85%) | 24 (15%) | 160 |
|  |  |  |  |  |
| 1718 | Negative | 124 | 8 | 132 (82.5%) |
|  | Positive | 7 | 21 | 28 (17.5%) |
|  | Total | 131 (81.9%) | 29 (18.1%) | 160 |
|  |  |  |  |  |
| 0092 | Negative | 132 | 0 | 132 (82.5%) |
|  | Positive | 8 | 20 | 28 (17.5%) |
|  | Total | 140 (87.5%) | 20 (12.5%) | 160 |
|  |  |  |  |  |
| 0612 | Negative | 127 | 5 | 132 (82.5%) |
|  | Positive | 9 | 19 | 28 (17.5%) |
|  | Total | 136 (85%) | 24 (15%) | 160 |
|  |  |  |  |  |
| 0937 | Negative | 128 | 4 | 132 (82.5%) |
|  | Positive | 16 | 12 | 28 (17.5%) |
|  | Total | 144 (90%) | 16 (10%) | 160 |
|  |  |  |  |  |
| 1910+1718 | Negative | 129 | 3 | 132 (82.5%) |
|  | Positive | 6 | 22 | 28 (17.5%) |
|  | Total | 135 (84.4%) | 25 (15.6%) | 160 |
|  |  |  |  |  |
| 1910+0092 | Negative | 130 | 2 | 132 (82.5%) |
|  | Positive | 7 | 21 | 28 (17.5%) |
|  | Total | 137 (85.6%) | 23 (14.4%) | 160 |
|  |  |  |  |  |
| 1910+0612 | Negative | 130 | 2 | 132 (82.5%) |
|  | Positive | 8 | 20 | 28 (17.5%) |
|  | Total | 138 (86.3%) | 22 (13.7%) | 160 |
|  |  |  |  |  |
| 1910+0937 | Negative | 129 | 3 | 132 (82.5%) |
|  | Positive | 8 | 20 | 28 (17.5%) |
|  | Total | 137 (85.6%) | 23 (14.4%) | 160 |
|  |  |  |  |  |
| 1718+0612 | Negative | 126 | 6 | 132 (82.5%) |
|  | Positive | 7 | 21 | 28 (17.5%) |
|  | Total | 133 (83.1%) | 27 (16.9%) | 160 |
|  |  |  |  |  |
| 1718+0937 | Negative | 128 | 4 | 132 (82.5%) |
|  | Positive | 7 | 21 | 28 (17.5%) |
|  | Total | 135 (84.4%) | 25 (15.6%) | 160 |
|  |  |  |  |  |
| 0092+0612 | Negative | 132 | 0 | 132 (82.5%) |
|  | Positive | 8 | 20 | 28 (17.5%) |
|  | Total | 140 (87.5%) | 20 (12.5%) | 160 |
|  |  |  |  |  |
| 0092+0937 | Negative | 132 | 0 | 132 (82.5%) |
|  | Positive | 8 | 20 | 28 (17.5%) |
|  | Total | 140 (87.5%) | 20 (12.5%) | 160 |
|  |  |  |  |  |
| 0612+0937 | Negative | 126 | 6 | 132 (82.5%) |
|  | Positive | 9 | 19 | 28 (17.5%) |
|  | Total | 135 (84.4%) | 25 (15.6%) | 160 |
|  |  |  |  |  |
| 1910+1718+0092 | Negative | 131 | 1 | 132 (82.5%) |
|  | Positive | 7 | 21 | 28 (17.5%) |
|  | Total | 138 (86.3%) | 22 (13.7%) | 160 |
|  |  |  |  |  |
| 1910+1718+0612 | Negative | 130 | 2 | 132 (82.5%) |
|  | Positive | 7 | 21 | 28 (17.5%) |
|  | Total | 137 (85.6%) | 23 (14.4%) | 160 |
|  |  |  |  |  |
| 1910+1718+0937 | Negative | 130 | 2 | 132 (82.5%) |
|  | Positive | 7 | 21 | 28 (17.5%) |
|  | Total | 137 (85.6%) | 23 (14.4%) | 160 |
|  |  |  |  |  |
| 1910+0092+0612 | Negative | 130 | 2 | 132 (82.5%) |
|  | Positive | 7 | 21 | 28 (17.5%) |
|  | Total | 137 (85.6%) | 23 (14.4%) | 160 |
|  |  |  |  |  |
| 1910+0092+0937 | Negative | 130 | 2 | 132 (82.5%) |
|  | Positive | 7 | 21 | 28 (17.5%) |
|  | Total | 137 (85.6%) | 23 (14.4%) | 160 |
|  |  |  |  |  |
| 1910+0612+0937 | Negative | 130 | 2 | 132 (82.5%) |
|  | Positive | 8 | 20 | 28 (17.5%) |
|  | Total | 138 (86.3%) | 22 (13.7%) | 160 |
|  |  |  |  |  |
| 1718+0092+0937 | Negative | 130 | 2 | 132 (82.5%) |
|  | Positive | 7 | 21 | 28 (17.5%) |
|  | Total | 137 (85.6%) | 23 (14.4%) | 160 |
|  |  |  |  |  |
| 1718+0612+0937 | Negative | 127 | 5 | 132 (82.5%) |
|  | Positive | 7 | 21 | 28 (17.5%) |
|  | Total | 134 (83.8%) | 26 (16.2%) | 160 |
|  |  |  |  |  |
| 0092+0612+0937 | Negative | 132 | 0 | 132 (82.5%) |
|  | Positive | 8 | 20 | 28 (17.5%) |
|  | Total | 140 (87.5%) | 20 (12.5%) | 160 |
|  |  |  |  |  |
| All except 1718 | Negative | 130 | 2 | 132 (82.5%) |
|  | Positive | 7 | 21 | 28 (17.5%) |
|  | Total | 137 (85.6%) | 23 (14.4%) | 160 |
|  |  |  |  |  |
| All except 0092 | Negative | 130 | 2 | 132 (82.5%) |
|  | Positive | 7 | 21 | 28 (17.5%) |
|  | Total | 137 (85.6%) | 23 (14.4%) | 160 |
|  |  |  |  |  |
| All except 0612 | Negative | 131 | 1 | 132 (82.5%) |
|  | Positive | 7 | 21 | 28 (17.5%) |
|  | Total | 138 (86.3%) | 22 (13.7%) | 160 |
|  |  |  |  |  |
| All except 0937 | Negative | 131 | 1 | 132 (82.5%) |
|  | Positive | 7 | 21 | 28 (17.5%) |
|  | Total | 138 (86.3%) | 22 (13.7%) | 160 |
|  |  |  |  |  |
| All proteins | Negative | 131 | 1 | 132 (82.5%) |
|  | Positive | 7 | 21 | 28 (17.5%) |
|  | Total | 138 (86.3%) | 22 (13.7%) | 160 |

**Supplementary** **Table 5:** True and False Negative, True and False Positive values as calculated based on the Binary logistic regression analysis for each protein in separate and for the combinations of the proteins. No results have been calculated for the combination of the proteins 1718+0092, 1718+0092+0612 and the combination of all the proteins without 1910 because the Hosmer and Lemeshow test failed. From the Table the number of samples agreeing with the clinical diagnosis and presenting with proteins above or below the cut-off set up for each one in the current study can be extracted.

|  |  |  |  |  |  |  |  |  |  |  |  |  | **Model build up** | | | |
| --- | --- | --- | --- | --- | --- | --- | --- | --- | --- | --- | --- | --- | --- | --- | --- | --- |
| **Protein/combinations of proteins** | **TPR** | **TNR** | **PPV** | **NPV** | **FPR** | **FNR** | **lr+** | **lr-** | **OR** | **Acc** | **pe** | **κ** | **B** | **Exp(B)** | | **Constant** |
| 1910 | 0,714 | 0,970 | 0,833 | 0,941 | 0,030 | 0,286 | 23,571 | 0,295 | 80,000 | 0,925 | 0,728 | 0,725 | 3,398 | 29,912 |  | -7,674 |
| 1718 | 0,750 | 0,939 | 0,724 | 0,947 | 0,061 | 0,250 | 12,375 | 0,266 | 46,500 | 0,906 | 0,707 | 0,680 | 2,009 | 7,453 |  | -5,259 |
| 0092 | 0,714 | 1,000 | 1,000 | 0,943 | 0,000 | 0,286 | 0,000 | 0,286 | 0,000 | 0,950 | 0,744 | 0,805 | 2,674 | 11,495 |  | -3,505 |
| 0612 | 0,679 | 0,962 | 0,792 | 0,934 | 0,038 | 0,321 | 17,914 | 0,334 | 53,622 | 0,913 | 0,728 | 0,679 | 1,776 | 5,904 |  | -3,421 |
| 0937 | 0,429 | 0,970 | 0,750 | 0,889 | 0,030 | 0,571 | 14,143 | 0,589 | 24,000 | 0,875 | 0,760 | 0,479 | 2,238 | 9,377 |  | -3,297 |
| 1910+1718 | 0,786 | 0,977 | 0,880 | 0,956 | 0,023 | 0,214 | 34,571 | 0,219 | 157,667 | 0,944 | 0,723 | 0,797 | 2,76 | 15,799 | [1910] | -7,928 |
| 1910+0092 | 0,750 | 0,985 | 0,913 | 0,949 | 0,015 | 0,250 | 49,500 | 0,254 | 195,000 | 0,944 | 0,732 | 0,790 | 2,541 | 12,696 | [1910] | -6,834 |
| 1910+0612 | 0,714 | 0,985 | 0,909 | 0,942 | 0,015 | 0,286 | 47,143 | 0,290 | 162,500 | 0,938 | 0,736 | 0,764 | 2,954 | 19,189 | [1910] | -7,603 |
| 1910+0937 | 0,714 | 0,977 | 0,870 | 0,942 | 0,023 | 0,286 | 31,429 | 0,292 | 107,500 | 0,931 | 0,732 | 0,744 | 3,058 | 21,28 | [1910] | -7,879 |
| 1718+0612 | 0,750 | 0,955 | 0,778 | 0,947 | 0,045 | 0,250 | 16,500 | 0,262 | 63,000 | 0,919 | 0,715 | 0,715 | 1,742 | 5,708 | [1718] | -5,247 |
| 1718+0937 | 0,750 | 0,970 | 0,840 | 0,948 | 0,030 | 0,250 | 24,750 | 0,258 | 96,000 | 0,931 | 0,723 | 0,751 | 1,76 | 5,815 | [1718] | -5,584 |
| 0092+0612 | 0,714 | 1,000 | 1,000 | 0,943 | 0,000 | 0,286 | 0,000 | 0,286 | 0,000 | 0,950 | 0,744 | 0,805 | 2,713 | 15,069 | [0092] | -3,481 |
| 0092+0937 | 0,714 | 1,000 | 1,000 | 0,943 | 0,000 | 0,286 | 0,000 | 0,286 | 0,000 | 0,950 | 0,744 | 0,805 | 2,787 | 16,228 | [0092] | -3,391 |
| 0612+0937 | 0,679 | 0,955 | 0,760 | 0,933 | 0,045 | 0,321 | 14,929 | 0,337 | 44,333 | 0,906 | 0,723 | 0,661 | 1,383 | 3,985 | [0612] | -3,644 |
| 1910+1718+0092 | 0,750 | 0,992 | 0,955 | 0,949 | 0,008 | 0,250 | 99,000 | 0,252 | 393,000 | 0,950 | 0,736 | 0,811 | 2,169 | 8,749 | [1910] | -7,091 |
| 1910+1718+0612 | 0,750 | 0,985 | 0,913 | 0,949 | 0,015 | 0,250 | 49,500 | 0,254 | 195,000 | 0,944 | 0,732 | 0,790 | 2,656 | 14,24 | [1910] | -7,869 |
| 1910+1718+0937 | 0,750 | 0,985 | 0,913 | 0,949 | 0,015 | 0,250 | 49,500 | 0,254 | 195,000 | 0,944 | 0,732 | 0,790 | 2,597 | 13,424 | [1910] | -8,278 |
| 1910+0092+0612 | 0,750 | 0,985 | 0,913 | 0,949 | 0,015 | 0,250 | 49,500 | 0,254 | 195,000 | 0,944 | 0,732 | 0,790 | 2,54 | 12,685 | [1910] | -6,863 |
| 1910+0092+0937 | 0,750 | 0,985 | 0,913 | 0,949 | 0,015 | 0,250 | 49,500 | 0,254 | 195,000 | 0,944 | 0,732 | 0,790 | 2,545 | 12,739 | [1910] | -7,002 |
| 1910+0612+0937 | 0,714 | 0,985 | 0,909 | 0,942 | 0,015 | 0,286 | 47,143 | 0,290 | 162,500 | 0,938 | 0,736 | 0,764 | 2,94 | 18,912 | [1910] | -7,66 |
| 1718+0092+0937 | 0,750 | 0,985 | 0,913 | 0,949 | 0,015 | 0,250 | 49,500 | 0,254 | 195,000 | 0,944 | 0,732 | 0,790 | 1,225 | 3,405 | [1718] | -4,547 |
| 1718+0612+0937 | 0,750 | 0,962 | 0,808 | 0,948 | 0,038 | 0,250 | 19,800 | 0,260 | 76,200 | 0,925 | 0,719 | 0,733 | 1,776 | 5,907 | [1718] | -5,599 |
| 0092+0612+0937 | 0,714 | 1,000 | 1,000 | 0,943 | 0,000 | 0,286 | 0,000 | 0,286 | 0,000 | 0,950 | 0,744 | 0,805 | 2,774 | 16,027 | [0092] | -3,392 |
| All except 1718 | 0,750 | 0,985 | 0,913 | 0,949 | 0,015 | 0,250 | 49,500 | 0,254 | 195,000 | 0,944 | 0,732 | 0,790 | 2,545 | 12,748 | [1910] | -6,997 |
| All except 0092 | 0,750 | 0,985 | 0,913 | 0,949 | 0,015 | 0,250 | 49,500 | 0,254 | 195,000 | 0,944 | 0,732 | 0,790 | 2,602 | 13,492 | [1910] | -8,196 |
| All except 0612 | 0,750 | 0,992 | 0,955 | 0,949 | 0,008 | 0,250 | 99,000 | 0,252 | 393,000 | 0,950 | 0,736 | 0,811 | 2,209 | 9,106 | [1910] | -7,369 |
| All except 0937 | 0,750 | 0,992 | 0,955 | 0,949 | 0,008 | 0,250 | 99,000 | 0,252 | 393,000 | 0,950 | 0,736 | 0,811 | 2,103 | 8,194 | [1910] | -6,882 |
| All proteins | 0,750 | 0,992 | 0,955 | 0,949 | 0,008 | 0,250 | 99,000 | 0,252 | 393,000 | 0,950 | 0,736 | 0,811 | 2,141 | 8,507 | [1910] | -7,405 |

**Supplementary** **Table 6:** Statistical factors associated with the comparison between the indirect immunofluorescence antibody test (IFAT) and the recombinant protein-based ELISA for the detection of serum phase I IgG antibodies against *C. burnetii*. The statistical analysis was performed based on Binary logistic regression. For the combination of the proteins 1718+0092, 1718+0092+0612 and the combination of all the proteins without 1910 the analysis failed to produce any results since the Hosmer and Lemeshow test failed. Column B indicates the factor that should be multiplied with the value of each protein as detected following the ELISA testing. Column Constant indicates the value that should be subtracted following the multiplication. The name inside the at the Exp(B) indicates the protein that the model kept following the analysis.
